# Octreotide-periplocymarin conjugate prodrug for improving targetability and anti-tumor efficiency: synthesis, *in vitro* and *in vivo* evaluation

**DOI:** 10.18632/oncotarget.13389

**Published:** 2016-11-16

**Authors:** Hui-Yun Zhang, Wen-Qian Xu, Yuan-yuan Zheng, Emmanuel Omari-Siaw, Yuan Zhu, Xia Cao, Shan-Shan Tong, Jiang-nan Yu, Xi-ming Xu

**Affiliations:** ^1^ Department of Pharmaceutics, School of Pharmacy, Center for Nano Drug/Gene Delivery and Tissue Engineering, Jiangsu University, Zhenjiang, People’s Republic of China; ^2^ School of Pharmacy, China Pharmaceutical University, Nanjing, People’s Republic of China

**Keywords:** cardiac glycosides, periplocymarin, octreotide, targetability, octreotide- periplocymarin conjugate

## Abstract

Cardiac glycosides could increase intracellular Ca^2+^ ion by inhibiting the Na^+^/K^+^ATPase to induce apoptosis in many tumor cells. However, narrow therapeutic index, poor tumor selectivity and severe cardiovascular toxicity hinder their applications in cancer treatment. To improve the safety profile and tumor targetablility of cardiac glycosides, we designed octreotide conjugated periplocymarin, a cardiac glycoside isolated from *Cortex periplocae*. The conjugate showed higher cytotoxicity on MCF-7 cells and HepG2 tumor cells (SSTRs overexpression) but much less toxicity in L-02 normal cells. Tissue distribution studies of the conjugate using H_22_ tumor model in mice showed higher accumulation in tumor and lower distribution in heart and liver than periplocymarin. Furthermore, *in vivo* anticancer effects of the conjugate on mice bearing H_22_ cancer xenografts confirmed enhanced anti-tumor efficacy and decreased systemic toxicity. Altogether, octreotide-conjugated periplocymarin demonstrated tumor selectivity and may be useful as a targeting agent to improve the safety profile of cardiac glycosides for cancer therapy.

## INTRODUCTION

Cardiac steroids (CS) or cardiotonic glycosides represent a group of compounds used clinically to increase cardiac contractile force in patients with congestive heart failure and cardiac arrhythmias [1, 2]. Recently, a number of publications have emphasized that apoptosis produced by cardiotonic glycosides in human tumor cells could be achieved at concentrations with no toxicity in humans, and therefore, these agents might be useful for cancer treatment [3, 4]. Over the last 10 years, interest in developing CS as anti-cancer agents has grown progressively. However, the classic CS appear to lack sufficient anti-tumor activity to be used as single anti-cancer agents at clinically acceptable doses [5].

Our laboratory has been able to screen periplocymarin with antitumor effect from *Cortex periplocae* by lipid-raft chromatography, which was found to be linked to the tyrosine kinase receptor [6]. It has been reported that periplocymarin strongly inhibited proliferation of PC3, U937, HCT-8, Bel-7402, BGC823, A549 and A2780 cell lines *in vitro* with IC_50_ values of 0.02–0.29 μM [7]. What is more, periplocymarin showed more cytotoxicity than the reference compounds (ouabain, periplogenin and periplocin) and could induce apoptosis in PC3 cells [8]. However, as a monosaccharide cardiac glycosides, its short elimination half-life, narrow therapeutic index, lack of tumor selectivity and severe adverse effects have hindered its wide applications in cancer treatment [9, 10]. Recently, targeted chemotherapy has become a novel approach to the treatment of cancers due to improved efficacy and reduced toxicity [11, 12].

Somatostatin (SST) is a neuropeptide that exerts powerful inhibitory action against several parts of the endocrine system [13]. The cellular actions of SST are inhibited by five specific somatostatin receptors subtypes (SSTR 1–5) which belong to the super-family of G-protein coupled receptors. SSTRs are widely distributed in the body, including normal cells like secretory cells, lymphocytes and tumor cells. However, most neuroendocrine tumors and their metastases express SSTRs to a much greater extent than normal tissues [14]. The tumors predominantly express SSTR-2, followed by SSTR-1, SSTR-5, SSTR-3 and SSTR-4 [15]. Whereas, the clinical usefulness of naturally occurring somatostatins is limited by its lack of SSTRs selectivity and short half-life in circulation (1–3 min). Therefore, synthetic derivatives including octreotide and lanreotide, have been created with improved metabolic stability and increased selectivity to SSTRs [16, 17].

Octreotide (OCT), an octapetide analogue with the same function as endogenous somatostatin, is found to be stronger and durable *in vivo* [18] with plasma half-life 30 times more than endogenous somatostatin [19]. Since octreotide mainly binds to SSTR-2, SSTR-3 and SSTR-5, it has been developed as a specific carrier to deliver antitumor drug into tumor cells via SSTR endocytosis and has been successfully applied in radio-oncology [20–23].

This study, therefore, designed periplocymarin conjugated with octreotide which was synthesized by coupling PPM–succinate to the amino-terminal end of octreotide. The present study investigated the basic physicochemical characteristics of these prodrugs. The cytotoxicity of OCT(Phe)-PPM was evaluated on breast cancer cells, MCF-7 and Hepatoma cells, HepG2, in which SSTR2 is overexpressed. Finally, *in vivo* biodistribution and therapeutic efficacy alongside systemic toxicity of the prodrugs were further evaluated in H_22_ tumor-bearing mice.

## RESULTS

### Preparation and characterization of periplocymarin from *Cortex periplocae*

The periplocymarin from *Cortex Periplocae* were prepared by a modified version of enzymatic hydrolysis method. The yield of periplocymarin was 0.081% and the results of the spectroscopic analyses were as follows: periplocymarin: ESI-MS (positive): m/z 535.35 [M-H]^-^; ^1^H-NMR ((CD_3_)_2_SO, 400 MHz) δ: 0.90 (3h, s, H-18), 0.95 (3 h, s, H-19), 3.21 (1H, dd, J = 3.2, 9.5 Hz, H-17), 4.18 (1H, m, H-3), 3.45 (3H, s, H-7′), 4.85 (1H, dd, J = 2.0, 9.6 Hz, H-1′), 4.95 (1H, dd, J = 1.6, 18.4Hz, H-21a), 5.05 (1H, dd, J = 1.6, 18.4Hz, H-21b), 5.92 (1H, s, H-22). ^13^C-NMR (400 MHz, CD3OD). δ:176.95(C-20), 175.81 (C-23), 116.45 (C-22), 96.79(C-1′), 84.89 (C-14), 75.75 (C-4′), 74.34 (C-5), 73.93 (C-3), 72.93(C-3′), 70.11 (C- 5′), 67.00 (C-21), 56.74 (C-7′), 50.53 (C-17), 49.48 (C-13), 40.42 (C-10), 40.23 (C-8), 39.49 (C-12), 38.77 (C-9), 37.14 (C-4), 34.39 (C-6), 34.11(C-2′), 31.95 (C-15), 26.60 (C-2), 25.37 (C-16), 25.16 (C-1), 23.35 (C-7), 21.30 (C- 11), 17.25 (C-6′), 15.84 (C-19), 14.93 (C-18). The MS and NMR information shown above indicated that we had successfully prepared purified periplocymarin from *Cortex Periplocae* [24].

### Synthesis of OCT(Phe)-S-PPM, OCT(Lys)-S-PPM and OCT-2S-2PPM

**Figure 1:**
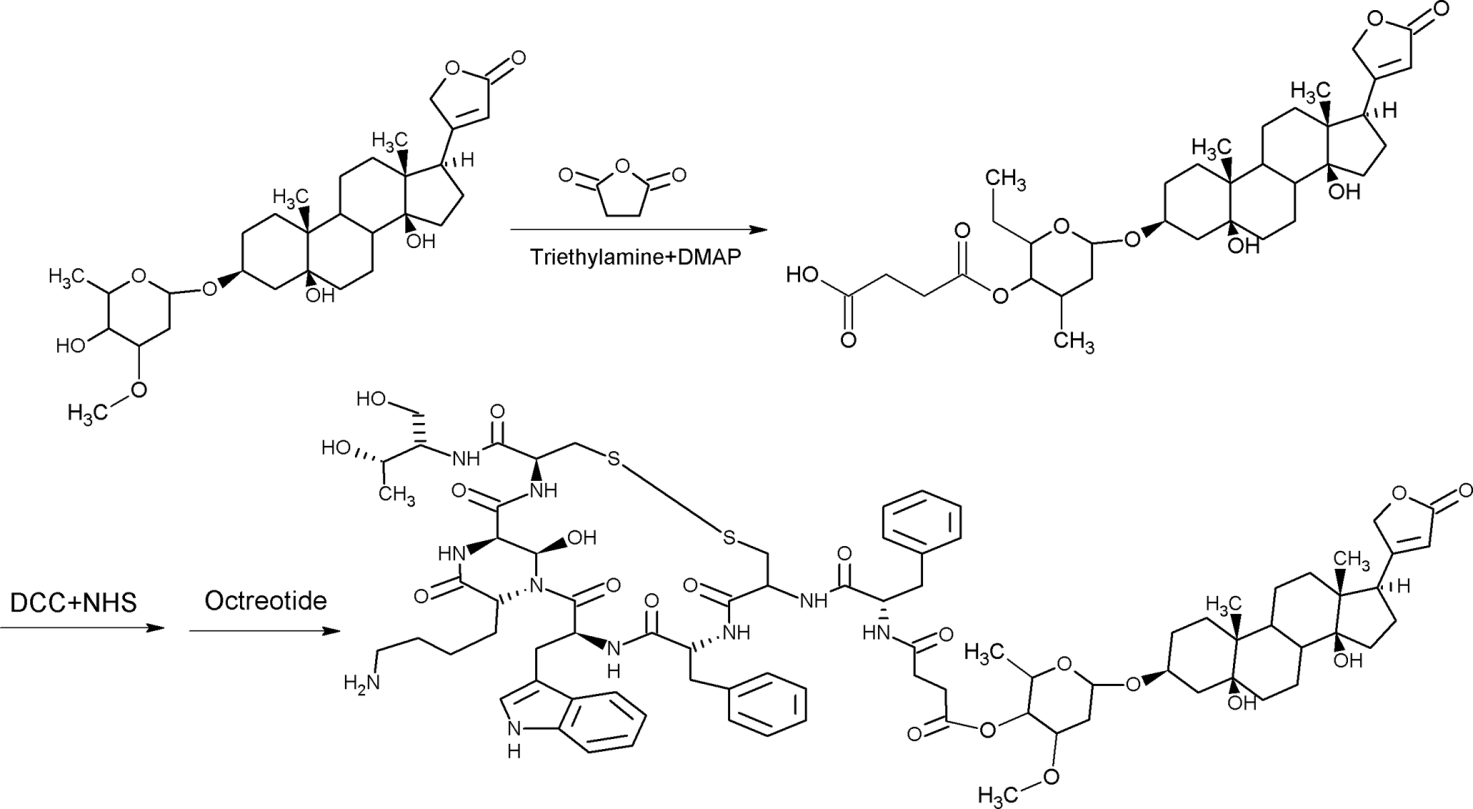
Synthetic route of OCT(Phe)-S-PPM PPM was modified with carboxyl group by reacting with succinic anhydride, the modified carboxyl was activated with hydroxysuccinimide (NHS), in the presence of DCC, to form an active ester group. Then the free amine groups (the N-terminal (Phe) on OCT) were crosslinked with the activated PPM to form OCT(Phe)-S-PPM.

Octreotide-periplocymarin conjugate (OCT-PPM) was synthesized by bifunctional crosslinking method as described in previous studies (Figure 1). The first step was to modify PPM with carboxyl group by reacting with succinic anhydride. The electrospray ionization mass spectrometry (ESI–MS) result gave [M+H]^+^ mass/charge (m/z) value of 635.45 Da, the ^1^H-NMR spectra yielded δ: 2.6 (t, succinic anhydride 2 CH_2_, 4H) and ^13^C-NMR spectra produced δ:174.82 (succinic anhydride, C-4), 171.68 (succinic anhydride, C-1), 29.012 (succinic anhydride, C-3), 28.72 (succinic anhydride, C-2). These results showed that SPPM was successfully synthesized.

**Figure 2:**
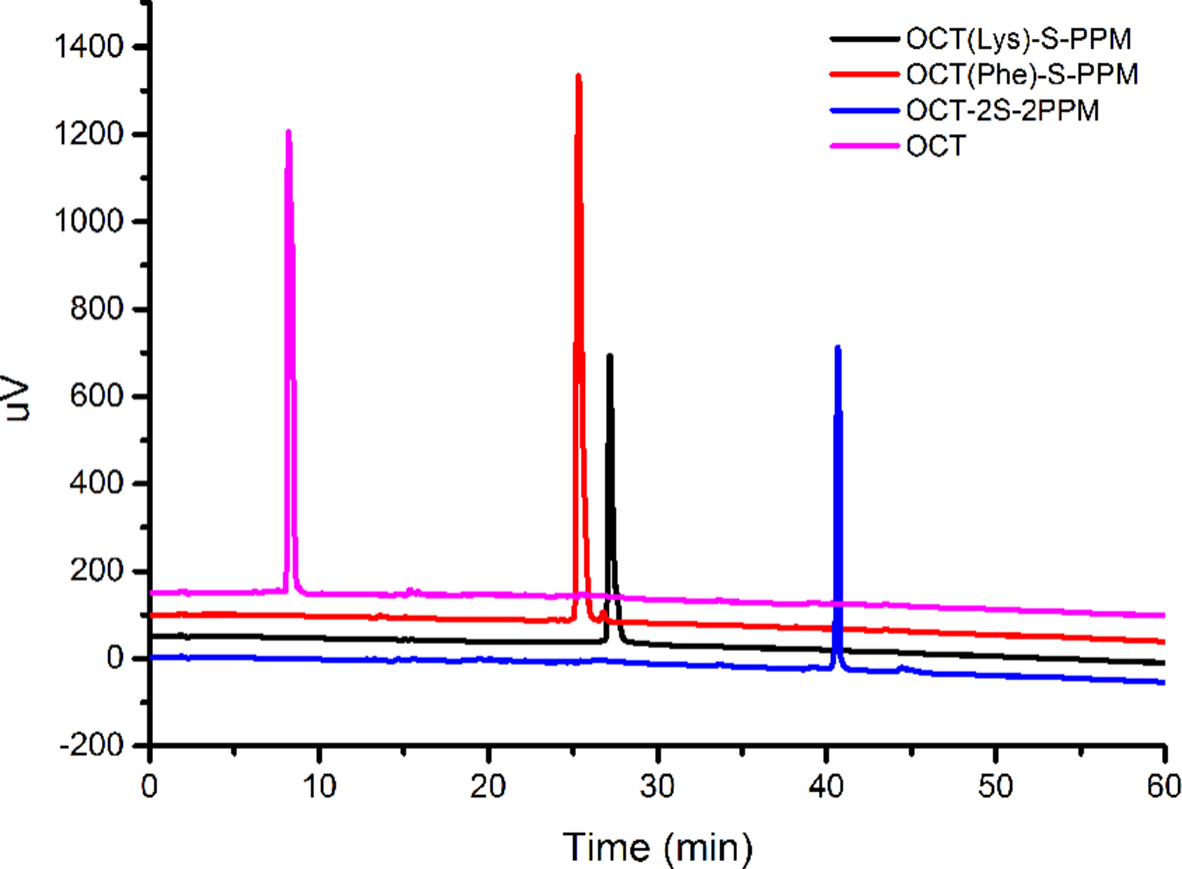
LC-UV analysis of octreotide conjugates

The next step was to activate SPPM with hydroxysuccinimide (NHS), in the presence of DCC, to form an active ester group. Then, the free amine groups (the N-terminal (Phe) and the lysine (Lys) residue) on octreotide were crosslinked with the activated PPM to form OCT(Phe)-S-PPM, OCT(Lys)-S-PPM and OCT-2S-2PPM. As shown in Figure 2A, the developed HPLC method effectively separated OCT(Phe)-S-PPM from OCT, OCT(Lys)-S-PPM and OCT-2S-2PPM.

**Figure 3:**
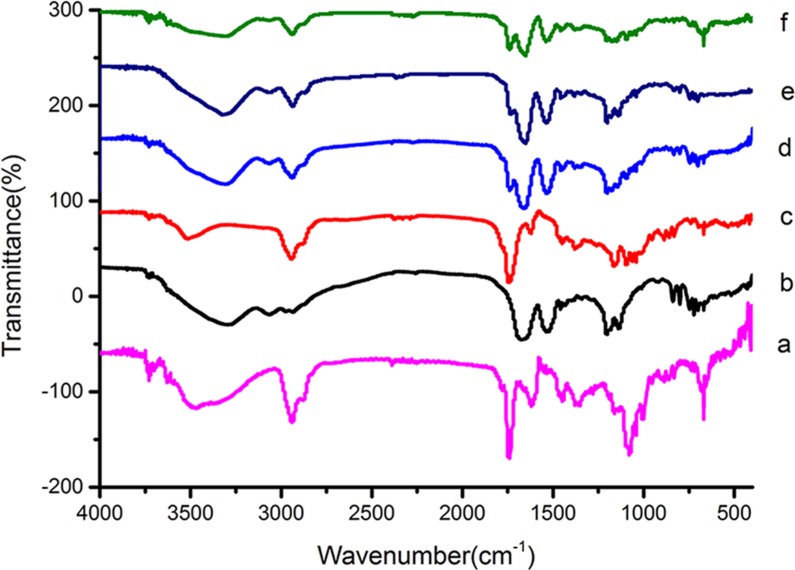
FTIR spectrum of PPM (a), OCT (b), SPP (c), OCT(Phe)–S-PPM (d), OCT(Lys)-S-PPM (e), OCT-2S-2PPM (f)

To characterize the synthesized conjugate, LC-UV, FTIR and ^1^H-NMR analyses were performed. The retention time (RT) for OCT(Phe)-S-PPM, OCT(Lys)-S-PPM and OCT-2S-2PPM on LC-UV, under the conditions defined in the Experimental Section, was 25.85 min, 27.19 min, and 40.66 min, respectively, with a purity of 95%. The retention time for OCT under the same gradient condition was 8.20 min (Figure 2). The DRIFT spectra of the PPM revealed the characteristic bands corresponding to –OH groups (3700–3000 cm^−1^), –CH_3_ group (2955, 2870 cm^−1^), bands of C=C stretching (1620 cm^−1^), C–H bending (1450, 1350), carbonyl –C = O stretching (1740 cm^−1^), and C–O stretching (1050–1250 cm^−1^). The OCT revealed the characteristic bands corresponding to –OH groups (3700–3000 cm^−1^), bands of C = C stretching (1670 cm^−1^), and =C-H bending vibration (800–1000 cm^−1^). Concerning OCT(Phe)-S-PPM, OCT(Lys)-S-PPM and OCT-2S-2PPM, all related spectra exhibited the characteristic bands of PPM and OCT (Figure 2). In the case of OCT-2S-2PPM, the DRIFT showed that the –C = O stretching (1740 cm^- 1^) was stronger than OCT(Phe)-S-PPM and OCT(Lys)-S-PPM (Figure 3).

**Figure 4:**
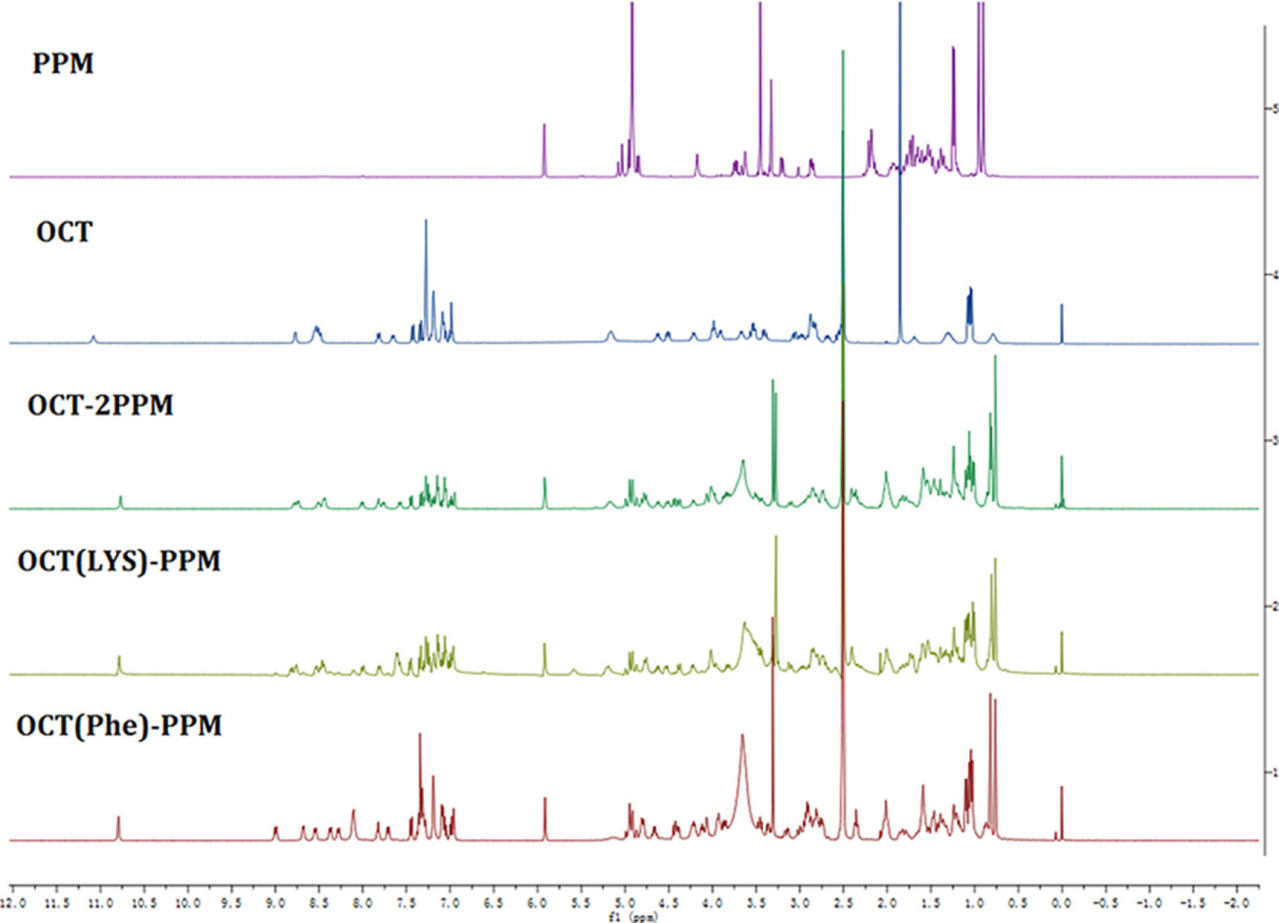
^1^H-NMR spectrum of OCT-PPM conjugates

The chemical structures of OCT(Phe)-S-PPM, OCT(Lys)-S-PPM and OCT-2S-PPM were confirmed by ^1^H NMR. As compared to the spectra of OCT and PPM, the characteristic peaks at 7.0∼7.5 ppm belong to the typical protons of OCT, and a specific peak at 7.28 PPM corresponds to N_ε_-H_2_ of Lys [25], while the characteristic peaks at 0.90, 0.95, 5.92 ppm belong to the typical protons of PPM. The ^1^H-NMR spectrum of OCT(Phe)-S-PPM, OCT(Lys)-S-PPM and OCT-2S-PPM are shown in Figure 4 alongside those of OCT and PPM.

### Cytotoxicity

**Figure 5:**
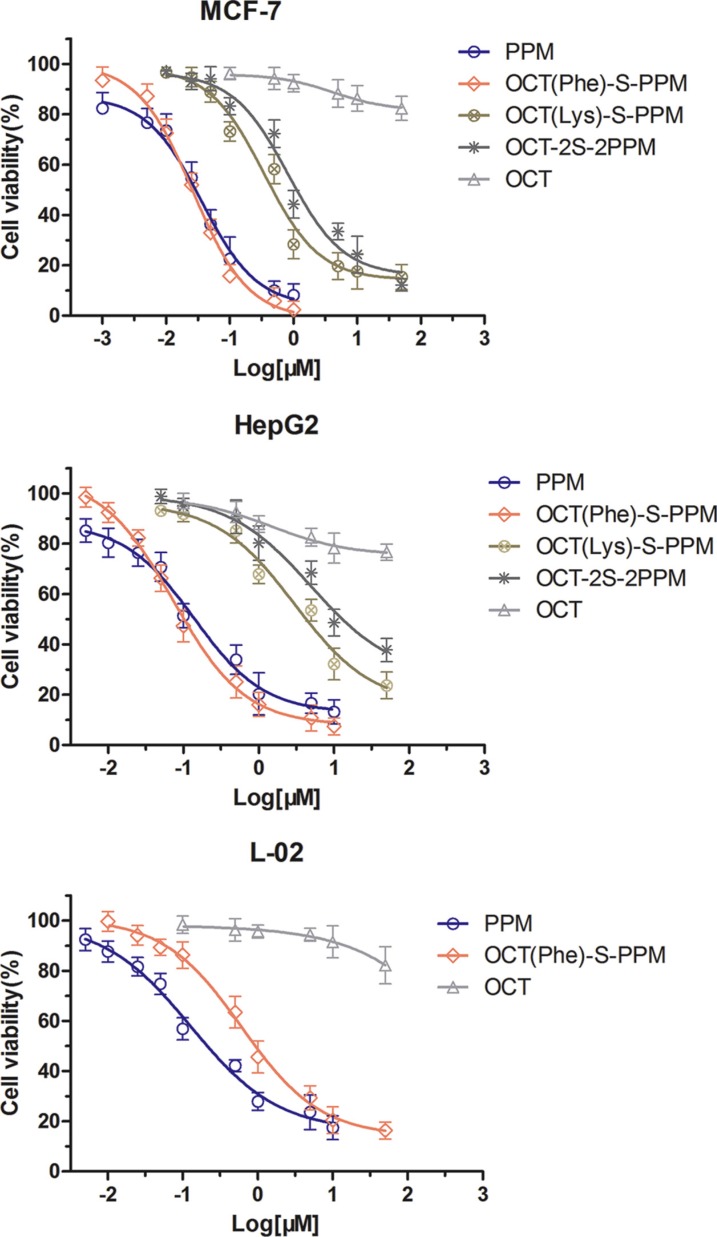
*In vitro* cytotoxicity of the peptide conjugation on to PPM with various concentration on HepG2, MCF-7 and L-02 cells after 72 h of incubation

The cytotoxicity of octreotide-periplocymarin conjugates (OCT-S-PPM) was evaluated on MCF-7, HepG2 cells and L-02 cells by the MTT assay using OCT and PPM as controls. The cell viability of different kinds of cell are shown in Figure 5. The IC_50_ values of different conjugates against the three cell lines are exhibited in Table 1. In MCF-7 and HepG2 cells, the strongest cytotoxicity among these conjugates was OCT(Phe)-S-PPM and its IC_50_ was lower than that of PPM. When conjugated with Phe-OCT, the cytotoxicity of PPM in MCF-7 (IC_50_ = 35.9 nM) and in HepG2 (IC_50_ = 137.6 nM) was enhanced 1.43 fold and 1.88 fold, respectively. As shown in Figure 5C, the cytotoxicity of OCT(Phe)-S-PPM to L-02 cells was significantly lower than that of PPM. *In vitro* data with dose-curve responses (in terms of growth inhibition) of PPM and its aglycone form on mouse versus human cancer cells was provided (Supplementary Figure S1 and Supplementary Figure S2). The IC_50_ values of PPM against the MFC murine gastric carcinoma cells was 38.40 nM, and lower than its aglycone form (IC_50_ = 508.9 nM).

**Table 1:** Improved *in vitro* cytotoxic activities of OCT(Phe)-S-PPM conjugates against some human cancer cells, IC_50_ (nM)

Compound	MCF-7		HepG2		L-02	
	IC_50_	95%CI	IC50	95%CI	IC_50_	95%CI
PPM	35.94	25.10–51.46	137.6	89.0–212.7	127.7	79.0–206.4
OCT(Phe)-S-PPM	26.26	20.42–33.78	73.1	53.8–99.4	609.8	434.4–856.4
OCT(Lys)-S-PPM	345.0	232.6–511.7	2984	1600–5568	NT	NT
OCT-2S-2PPM	820.7	564.2–119.4	4979	2035–12180	NT	NT
OCT	> 50 μM	NA	> 50 μM	NA	> 50 μM	NA

NT: not test. NA: not analysis

### Biodistribution in mouse sarcoma H_22_ cell tumor bearing mice

**Figure 6:**
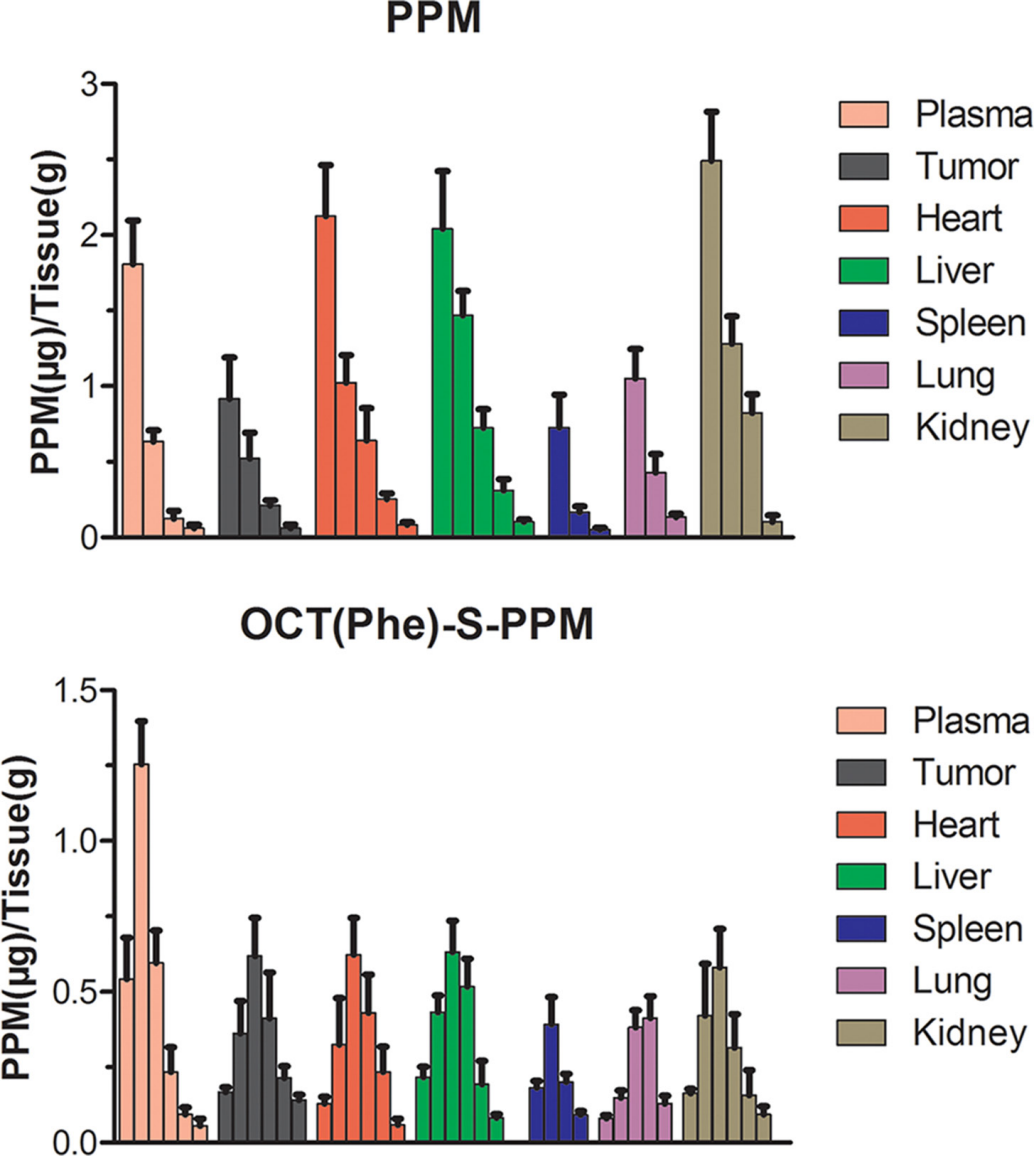
Tissue distribution of PPM after intravenous administration of PPM and OCT(Phe)-S-PPM injection in H22-bearing mice in blood, tumor, liver, spleen, lung, kidney and heart (error bars are mean ± SD, *n* = 3)

In this study, the *in vivo* biodistribution of PPM and OCT(Phe)-S-PPM following intravenous administration in H_22_ tumor bearing mice was investigated. Mice were sacrificed at 0.25, 0.50, 1, 2, 3 and 4 h, post injection and tissues were harvested to determine the level of PPM. The biodistribution of the two drugs in various organs (tumor, heart, liver, spleen, kidney and lung) was wide and rapid (Figure 6). The PPM distribution followed the order: Liver>Kidney>Heart>Tumor>Lung>Spleen. The OCT(Phe)-S-PPM distribution followed a different order: Liver>Tumor> Heart>Kidney>Lung>Spleen (Table 2).

**Table 2:** AUC_0–t_ values in various tissues after i.v. administration of OCT(Phe)-S-PPM and PPM injection at a dose of 4 mg/kg into H_22_ tumor-bearing mice.^a^

Tissue	OCT(Phe)-S-PPM (μg·h/g)	PPM (μg·h/g)
Plasma	1.409 ± 0.196*	0.812 ± 0.143
Heart	1.315 ± 0.239*	1.692 ± 0.283
Liver	1.441 ± 0.202*	1.967 ± 0.321
Spleen	0.632 ± 0.079*	0.257 ± 0.086
Lung	0.839 ± 0.083*	0.457 ± 0.092
Kidney	1.152 ± 0.132*	1.773 ± 0.393
Tumor	1.338 ± 0.195*	0.614 ± 0.128

a Data are given as mean ± SD (*n* = 5).

* *P* < 0.05, vs PPM injection.

At 15 min post injection (Figure 6A), PPM was rapidly distributed to liver, heart, tumor, kidney and spleen, with much accumulation in the liver, kidney and heart. The concentration of PPM in each tissue decreased sharply with time. What is more, the high distribution rate in the kidney indicated rapid clearance of PPM *in vivo*. The ratio of the PPM concentration in heart and blood was 1.18 at 15 min post injection, however it increased to 5.21 at 1 h post injection. This phenomenon also occurred in the liver.

Fortunately, the biodistribution and circulation time of OCT(Phe)-S-PPM was greatly improved for PPM (Figure 6B). In addition, the C_max_ in each tissue of OCT(Phe)-S-PPM was significantly lower than PPM. Comparing the area under the concentration–time curves (AUC_0–t_) in plasma, OCT(Phe)-S-PPM was 1.73-fold higher than that of PPM. The concentration of OCT(Phe)-S-PPM in tumor tissue was only 0.168 μg/g at 15 min post injection, which was lower than PPM (0.917 μg/g). However, OCT(Phe)-S-PPM concentration kept increasing till it reached the maximum concentration (0.618 μg/g) at 1 h. Compared with PPM, the plasma and tissue PPM concentration of OCT(Phe)-S-PPM showed a delayed profile. The AUC_0–t_ of OCT(Phe)-S-PPM (1.338 μg·h/g) in tumor was 2.17 fold higher than that of PPM (0.614 μg·h/g). What is more, with regards to the total AUC_0∼t_ of heart and liver, OCT(Phe)-S-PPM was 0.77- and 0.73- fold lower than that of PPM (*P* < 0.01), respectively. Statistical analysis shown in Table 2 indicated that PPM modified with OCT remarkably increased accumulation of PPM in H_22_ tumors (*P* < 0.05).

### Anti-tumor efficacy and toxicity of OCT(Phe)-S-PPM

**Figure 7:**
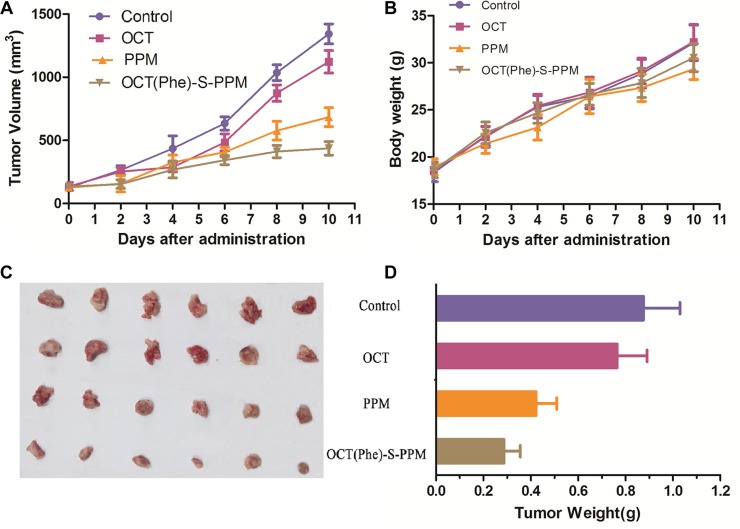
Graph showing tumor volume of H_22_ tumor-bearing mice in different treatment groups (**A**) antitumor effect in terms of tumor growth (error bars are mean ± SD, *n* = 10); (**B**) the change of body weight during the treatments; (**C**) tumor growth after systemic application of different treatment groups; (**D**) tumor weight (error bars are mean ± SD, *n* = 5)

To determine whether OCT(Phe)-S-PPM had better anti-tumor effect than PPM *in vivo* as we proposed, Kunming mice bearing H_22_ tumor were randomly divided into four groups and treated with control, PPM (4 mg/kg), OCT or OCT(Phe)-S-PPM (equimolar dose of PPM). The average tumor volume is shown in Figure 7A and 7B. An enhanced tumor inhibition effect was observed in the drug treatment groups. Since octreotide could deliver anti-tumor drugs to tumor via receptor-mediated targeting, the conjugate OCT(Phe)-S-PPM significantly reduced tumor volume by an average 1.56- fold compared to PPM, which was consistent with observations in cell tests and tissue distribution.

**Figure 8:**
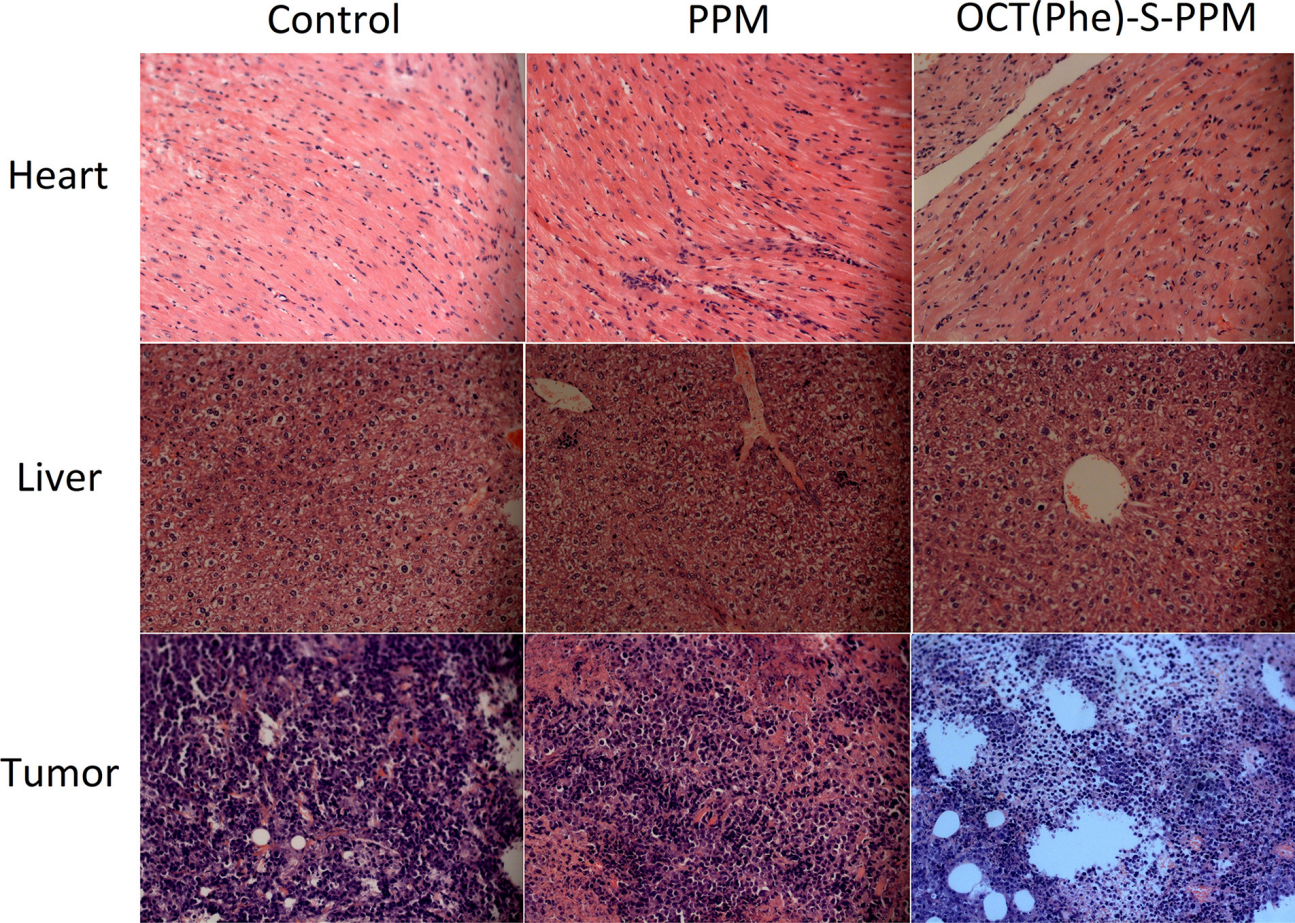
Typical histopathologic images of rat hearts, livers and tumors after treatment in H_22_-bearing mice with PPM and OCT(Phe)-S-PPM for ten days (HE staining, ×20)

The final tumor weight was measured and exhibited in Figure 7C and 7D. There was slight difference in tumor weight between control group (0.876 ± 0.154 g) and OCT group (0.765 ± 0.126 g). Additionally, the weight of tumor in PPM and OCT(Phe)-S-PPM group was 0.423 ± 0.087 g and 0.287 ± 0.069 g, respectively, which were much lower than that of control group (*P* < 0.01). The tumor inhibition rate of OCT(Phe)-S-PPM (67.23%) was significantly higher than that of PPM treatment (51.71%, *P* < 0.01). Furthermore, according to the histopathological examination of the tumors under a microscope (Figure 8), closely spaced tumor cells with hyperchromatic nuclei could be clearly observed in the control group, which demonstrates the rapid growth of the tumor cells. However, the tumor cells in the PPM and OCT(Phe)-S-PPM groups exhibited marked degeneration, disruption, and death, especially in the OCT(Phe)-S-PPM group. Hence, the i.v. administration of OCT(Phe)-S-PPM resulted in a significant inhibition of tumor growth. Altogether, it was evident that the antitumor efficacy of OCT(Phe)-S-PPM was greatly superior to that of PPM in H_22_ tumor bearing mice model.

From Figure 7B, the average body weight of PPM group was less than that of the control group (*P* < 0.01). Representative pathological images of hearts and livers are presented in Figure 8. For the PPM group, the liver sections exhibited obvious soma shrinking, dissociation of hepatic cord, dilation of hepatic sinusoids and lymphocytosis, while the heart sections exhibited irregularly arranged myocardial cells and slightly lymphocyte infiltration in the interstitial tissue of the myocardium, without any obvious myocardial degeneration and necrosis. Concerning the OCT(Phe)-S-PPM group, there were no evident differences from the control group. Additionally, the levels of hepatic function markers (AST, ALT, and ALP) in serum from prodrug treated groups induced negligible changes as compared with those from control group (Table 3). In contrast, the levels of these markers in serum from PPM group were significantly increased over control group. These results indicated that periplocymarin had severe side effects including hepatic and heart toxicity. However, OCT(Phe)-S-PPM reduced the toxic effects of PPM on the heart and liver.

**Table 3:** AST, ALT and ALP lever in the serum of mice sacrificed 24 h after the last Administration.^a^

Groups	ALT(U/L)	AST(U/L)	ALP(U/L)
Control	26.86 ± 6.44	50.55 ± 8.82	112.06 ± 14.29
OCT(Phe)-S-PPM	23.25 ±5.76	56.83 ± 7.34	124.90 ± 27.23
PPM	62.43 ± 13.52*	68.21 ± 8.95*	184.23 ± 21.48*
OCT	34.48 ± 6.28	51.25 ± 5.2	129.48 ± 18.54

a Data are given as mean ± SD (*n* = 5).

* *P* < 0.05, vs control injection.

## DISCUSSION

Chemically, glycosylated CSs are compounds presenting a steroid nucleus with a lactone moiety at position C_17_ and a sugar moiety at position C_3_. Cardiac glycosides can be divided into two types: the cardenolides (with an unsaturated butyrolactone ring) and the bufadienolides (with an α-pyrone ring). However, cardenolides and bufadienolides display subtle but important differences in terms of anticancer activity [26]. From the point of structure-activity relationship, glycosylated cardenolides show stronger cytotoxicity than its aglycone form while glycosylated bufadienolides display lower cytotoxicity than its aglycone form [27]. In agreement with numerous data published in literature, PPM displayed stronger *in vitro* growth inhibition than its aglycone form (periplogenin) on the three cell lines (Supplementary Table S1 and Figure S1). Although cardiac glycosides have potential effects on cancer, at present, evidence supporting their usefulness is still needed, and the safety profile of cardiac glycosides as anticancer agents must be considered [28].

Furthermore, the inherent high toxicity and poor therapeutic margin of these commonly used CS have prevented the development of cardiac glycoside as anti-cancer agents [2, 29]. The solution to the problem of cardiac glycoside toxicity mainly include structure modification, nanotechnology and use of prodrug. As widely reported, the absence of linked sugar units in structure–activity relationship could be crucial in increasing the antiproliferative effect of cardiotonic steroids [30, 31]. It means that conjugating a molecule to the single sugar unit of periplocymarin could reduce the cytotoxicity. However, OCT(Phe)-S-PPM exhibited significant cytotoxicity in HepG2 cells and MCF-7 cells (SSTRs overexpression) (Figure 5A and 5B) [32]. This could be explained by the increased cell internalization of OCT(Phe)-S-PPM owing to the high binding affinity of OCT to SSTRs. In addition, the lower cytotoxicity of OCT(Lys)-S-PPM and OCT-2S-2PPM demonstrated that periplocymarin conjugated with OCT at Lys-NH_2_ reduce the binding affinity of OCT with MCF-7 and HepG2 cells. On the other side, OCT(Phe)-S-PPM could reduce the cytotoxicity of PPM on normal cells. As previously reported, octreotide peptide has low cytotoxicity on normal cells that express low levels of SSTRs (e.g.L-02 cells) [33]. According to the previous report, murine cancer cells are much less sensitive to cardiotonic steroids than human cancer cells due to the fifth transmembrane part of the NAK-alpha-1 subunit that is twice mutated in mice [26, 27]. However, the results of cytotoxicity on mouse cancer cell showed that PPM was sensitive to MFC murine gasric cancer cell as well as MCF-7 human breast cancer cells. It means that PPM not only target the fifth transmembrane part of the NAK-alpha-1 subunit, also had some other target of blocking tumor growth [34, 35].

The biodistribution of PPM in mouse sarcoma H_22_ cell tumor bearing mice showed high distribution rate in the liver and heart which was the main cause for hepatotoxicity and cardiotoxicity [5, 36]. Overall, the concentration of PPM in tumor was comparable to other tissues, indicating poor tumor targeting [37]. However, OCT(Phe)-S-PPM modification could decrease the accumulation of PPM in heart and liver. The histopathological and hepatic function markers (AST, ALT, and ALP) in serum evaluation revealed that OCT(Phe)-S-PPM could reduce the toxicity of PPM on rat heart and liver. What is more, OCT(Phe)-S-PPM could enhance the drug retention time and increase concentration in tumor tissue via octreotide-SSTRs interaction on the SSTRs positive tumor.

There is no doubt that the short elimination half-life, rapid clearance and lack of tumor targeting could decrease the anti-tumor effectiveness of PPM. However, after conjugating with OCT, PPM exhibited a longer-circulating effect *in vivo* and higher accumulation in tumors via octreotide-SSTRs mediated active targeting. *In vivo* study, OCT(Phe)-S-PPM, benefiting from somatostatin mediated tumor targeting, showed markedly improved therapeutic effects compared to free periplocymarin.

The results of cytotoxicity assay *in vitro* and antitumor efficacy *in vivo* indicated that the conjugation to PPM by the polypeptide which specifically binds to the tumor-specific antigen or a peptide transporter that is over-expressed in cancer cells, could enhance the antitumor activity of the parent drug. The reasons are that uptake of octreotide-conjugated PPM was by the endocytosis of SSTRs and could accumulate in tumor tissues while PPM was transported across membranes via passive diffusion [9, 10]. Based on these findings, we thus anticipate the contribution of octreotide-conjugated antitumor drugs to the development of cardiac glycosides in clinical application.

## MATERIALS AND METHODS

### Materials

OCT was purchased from HuaJin Pharmaceutical Co., Ltd. (HangZhou, China). Bufalin was obtained from J&K scientific co., Ltd, (Beijing, China). Dicyclohexylcarbodiimide (DCC), N-Hydroxysuccinimide (NHS) and 4-(2- hydroxyethyl)-1-piperazineethanesulfonic acid (HEPES) were obtained from Aladdin Industrial Corporation (ShangHai, China). (3-(4,5-Dimethylthiazol-2-yl)-2,5- diphenyltetrazolium bromide (MTT) and trypsin were purchased from Beyotime Institute of Biotechnology (Jiangsu, China). Fetal bovine serum and Dulbecco’s modified Eagle’s medium (DMEM) were purchased from Gibco Company (Grand Island, NY). Chromatographically pure methanol and acetonitrile were obtained from Hanbon Technology Co., Ltd. (Jiangsu, China).

### Preparation of periplocymarin from *Cortex Periplocae*

Periplocymarin was prepared by a modified version of enzymatic hydrolysis method as described in previous studies with some modifications. Briefly, the dry *Cortex Periplocae* were extracted with water. The water extraction was concentrated and 85% ethanol was added to remove the insoluble parts. The ethanol solution was further concentrated to remove ethanol and extracted thrice with diethyl ether, ethyl acetate and n-butyl alcohol, successively. The n-butyl alcohol extraction (10.0 g) was enzymatically hydrolyzed with 0.6% helicasein citric acid and sodium citrate buffer (pH = 5.5, 100 mL) at 50°C for 24 h. The aqueous residue was extracted with dichloromethane-methanol and washed with water. After removal of the solvent, the residue was purified by means of C_18_ column chromatography to yield compound 1 (164 mg). Compound 1 was identified as periplocymarin by spectroscopic analyses and compared with published data.

### Synthesis of suc-periplocymarin (SPPM)

Periplocymarin (27.1 m, 0.05 mmol) and succinic anhydride (30 mg, 0.3 mmol) were dissolved in 3 mL of anhydrous methylene chloride (CH_2_Cl_2_) and stirred at room temperature for 24 h, followed by the addition of appropriate amount of triethylamine and 4-dimethyl-aminopyridine (DMAP). At the end of the reaction, the solution was evaporated to dryness under vacuum, the crude SPPM obtained was further purified by C_8_ column chromatography to afford 26.0 mg (80.8% yield) SPPM as a pale white solid.

### Synthesis of NHS activated PPM(NHS-PPM)

SPPM (128 mg, 0.2 mmol), DCC (77.9 mg, 0.408 mmol, 2 × excess), NHS (50.0 mg, 0.408 mmol, 2×excess) and 20 ml of dichloromethane were added to a round-bottom flask equipped with a magnetic stirring bar, attached to a nitrogen line and a bubbler. The reaction was maintained at room temperature for 24 h. The resulting mixture was filtered to remove N, N-dicyclohexylurea and the filtrate was dried under vacuum. The residue was purified using silica gel column chromatography, eluting with a dichloromethane-methanol solution of gradually increasing methanol content. The elution solvent was removed in a vacuum to give 90 mg of NHS-PPM with the total yield of 60.1%.

### 2.5. Synthesis of OCT(Phe)-S-PPM, OCT(Lys)-S-PPM and OCT-2S-2PPM

NHS-PPM (7.4 mg, 0.01 mmol) in 50% acetonitrile-0.1 M HEPES together with OCT (20.3 mg, 0.021 mmol, 2 × excess) was stirred in an ice-water bath, and adjusted to pH 8.4 with N-methylmorpholine. The solution was stirred further for 24 h and purified by C_18_ column chromatography eluting with acetonitrile/0.05% Formic acid-H_2_O (20:60 to 90:30 v/v gradient). The fractions containing the OCT conjugate were collected and lyophilized.

### Characterization of OCT(Phe)-S-PPM, OCT(Lys)-S-PPM and OCT-2S-2PPM

The characterization of the product was confirmed by LC-UV, FTIR and ^1^H-NMR. HPLC system (Shimadzu, Kyoto, Japan) was equipped with a quaternary pump, a Surveyor AS autosampler and a vacuum degasser. The chromatographic separation was performed on a Symmetric C_18_ column (5 μm, 4.6 × 150 mm, Waters, Milford, MA, USA) maintained at 30^°^C. The flow was 0.8 mL/min, with the mobile phase starting from 20% solvent A (0.05% TFA in water) and 90% solvent B (0.05% TFA in acetonitrile) at 0.01 min to 50% solvent A and 50% solvent B at 30 min, then to 5% solvent A and 95 % solvent B at 60 min; the sample injection volume was 20 μL. Infrared absorption spectra of the products were examined using DRIFT spectroscopy (Spectrum GX spectrophotometer, Perkin-Elmer, MA, USA) with a diffuse reflectance accessory (Pike Technology model). Each of the samples (2.4 mg) was mixed with 97.6 mg KBr and dried in an oven for 24 h at 135°C to keep the samples dry. The instrument was operated at a resolution of 4 cm^−1^ and 32 scans for each sample. The absorbency scans were analyzed between 400 and 4000 cm^−1^ for changes in the intensity of the sample peaks

^1^H-NMR spectra were recorded on a 400 MHz spectrometer (Bruker AVANCE, Switzerland) at room temperature in deuterated dimethyl sulfoxide with tetramethylsilane (TMS) as an internal standard and the chemical shifts were in ppm down field.

### Cell culture and growth inhibition assay

To test the antitumor activities of the synthesized compounds, we evaluated antiproliferative activities of these compounds against human cancer cell lines (Breast cancer cells, MCF-7; Hepatoma cells, HepG2) and human normal cell line (Liver cells, L-02), using the MTT assay as reported in previous studies [38]. Both cell lines were obtained from the Cell Bank of Academy of Science (Shanghai, China).

In brief, the cells were seeded into a 96-well plate at a density of 2.5 × 10^4^ cells/well and incubated with OCT modified PPMs containing various concentrations (ranging from 0.001 to 50.0 μM) for 72 h. After incubation, 20 μL of MTT (5 mg/mL in pH 7.4 PBS) was added to each well, and the plates further incubated at 37°C for 4 h. The medium was then replaced with 100 μL DMSO. The optical density was measured by Microplate Reader (Thermo Electron Corporation) at 595 nm. The cell inhibition rate (IR) was calculated as follows: IR (%) = 1−A/A_1_×100%, where A refers to the average absorbance intensity of treated group and A_1_ refers to average absorbance intensity of untreated control group (the vehicle control group).

### Tissue distribution in H_22_ cell tumor bearing mice

Male Kunming strain mice (KM, 18–22 g) were purchased from Qinglongshan, Experimental Animal Center (ZhenJiang, China). Animal experiments were conducted under principles of good laboratory animal care, and approved by the Ethical Committee for Laboratory Animals Care and Use of Jiangsu University. Mouse sarcoma (H_22_) cells were purchased from the Cell Bank of Academy of Science (Shanghai, China).

Approximately 1 × 10^7^ H_22_ cells suspended in PBS (pH 7.4) were inoculated subcutaneously into the right hind leg. When the tumor volume reached 1000 mm^3^ (around 7 days), mice were intravenously injected with PPM (at a dose of 4 mg/kg) or its equivalent OCT(Phe)-S-PPM via the tail vein (*n* = 5). At indicated time periods (0.16, 0.50, 1, 2, 3 and 4 h) after injection, blood samples were collected and mice were then sacrificed by cervical dislocation to obtain the heart, liver, spleen, lung, kidney and tumor tissues. All the samples were then washed with ice-cold saline to remove the excess fluid, weighed and stored at –20°C. The thawed tissue was homogenized in 0.9% sodium chloride solution to obtain 0.2 g/mL tissues homogenate. This was followed by the addition of bufalin (50 μl, 5 μg/ml) to the tissues (0.2 ml) with uniform mixing. Ethyl acetate (1.2 ml) was then added to the resulting mixture and thoroughly mixed for 5 min. The total supernatant or organic layer was separated by centrifugation at 3000 rpm for 10 min and transferred to a clean tube. The supernatant was dried with nitrogen at 40°C on water bath to obtain the residue, which was later reconstituted in a 0.2 mL of mobile phase solution. After centrifugation at 20,000 rpm for 10 min, the supernatant was analyzed by HPLC. The mobile phase consisted of acetonitrile and water (38:62, v/v, pH = 3.0, adjusted by H_3_PO_4_). The flow rate was 1.0 mL·min^-1^ and the detection wavelength was 220 nm. Other conditions included injection volume (20 μl), column temperature (25°C) and detection sensitivity (0.02 AUFS). The retention time of PPM and Bufalin was about 7.2 and 10.8 min, respectively. The calibration curve was A = 0.9958C-0.0299 (A represented the peak area ratio of PPM and Bufalin, while C was the concentration of PPM) and was linear in the range of 0.05–10 μg/mL with a correlation coefficient of R = 0.9995.

### Anti-tumor efficacy and toxicity

Treatment was initiated when the tumor diameter reached about 0.5 cm (around 3 days). Mice were randomly assigned to four groups (*n* = 6) namely the control, PPM, OCT and OCT(Phe)-S-PPM groups. Treatment were administered via tail vein at a dose of 4 mg/kg every other day during the experimental period for 5 times. The control group received corresponding amounts of blank injection (ethanol/Cremophor EL, v/v, 1/1, diluted 20 times with normal saline before the injection). Their weights were measured daily and tumor size was measured (major and minor axis) with a vernier caliper thereafter and at the end of the experiment. Tumor volumes were calculated using the formula: a^2^×b×0.52, where a and b refer to the longest and shortest diameter, respectively. Tumor tissues were removed from the sacrificed mice and weighed to calculate the tumor inhibition rate (TIR (%) = (1-Wt/Wc) × 100%, where Wt and Wc are the mean tumor weights of the treated groups and the negative control group, respectively. Moreover, the heart, liver and tumor were rinsed with normal saline, fixed in 10% formalin, and then embedded in paraffin blocks for later slicing and staining with hematoxylin and eosin (H&E). Finally, the sections were observed under a microscope for histopathological evaluations

### Statistical analysis

Statistical significance of differences between treatment groups in the biodistribution and anti-tumor effect were assessed using an unpaired Student’s *t-test*. A *p-value* of less than 0.05 was considered significant. 50% inhibiting concentration (IC_50_) of the sample was calculated with the aid of Curve-Expert software (version 1.3; Daniel G. Hyams, Hixson, TN, USA).

## SUPPLEMENTARY MATERIALS TABLE AND FIGURES


