# Supplementary material for: Octreotide-periplocymarin conjugate prodrug for improving targetability and anti-tumor efficiency: synthesis, in vitro and in vivo evaluation
Source: Oncotarget. 2016 Nov 16;7(52):86326–38. doi: 10.18632/oncotarget.13389 (PMC5349917; doi:10.18632/oncotarget.13389)
Supplement: Supplementary file 1 [file oncotarget-07-86326-s001.pdf]

Octreotide-periplocymarin conjugate prodrug for improving targetability and anti-tumor efficiency: synthesis, *in vitro* and *in vivo* evaluation

Supplementary Materials

Supplementary Table S1: *In vitro* cytotoxic activities of periplogenin against some human cancer cells, IC_{50} (nM)

Compound	MCF-7		HepG2		L-02		MFC	
	IC_{50}	95%CI	IC_{50}	95%CI	IC_{50}	95%CI	IC_{50}	95%CI
periplogenin	901.5	762.1–1066	580.5	363.5–927.0	406.3	315.3–523.7	508.9	424.1–610.7

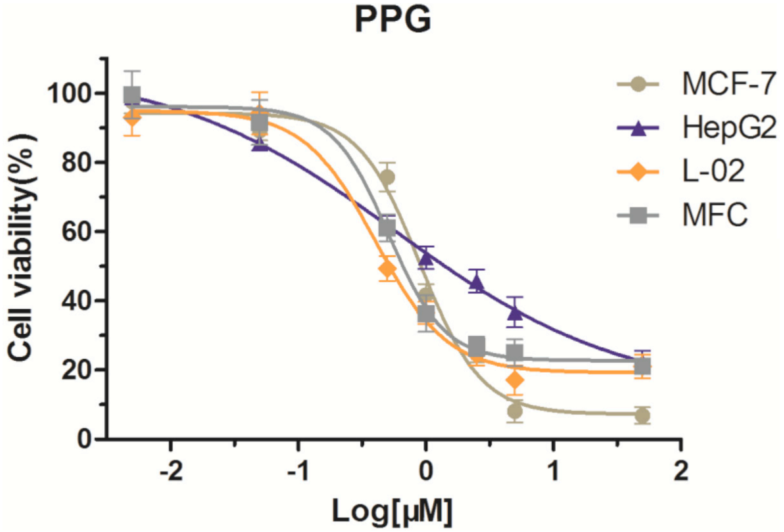

Supplementary Figure S1: *In vitro* cytotoxicity of periplogenin with various concentration on HepG-2 , MCF-7, MFC and L-02 cells after 72 h of incubation.

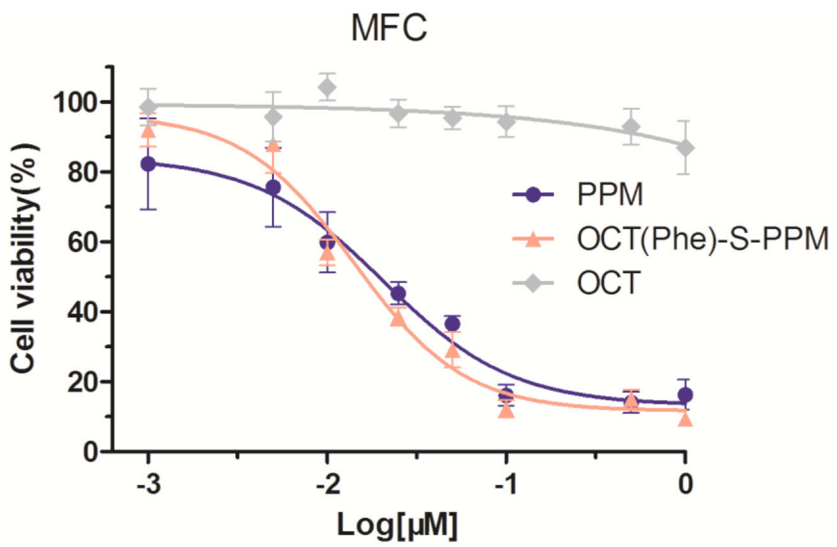

Supplementary Figure S2: ***In vitro*** **cytotoxicity of the peptide conjugation on to PPM with various concentration on MFC cells after 72 h of incubation.**
